# Supplementary material for: A gene prioritization method based on a swine multi-omics knowledgebase and a deep learning model
Source: Commun Biol. 2020 Sep 10;3:502. doi: 10.1038/s42003-020-01233-4 (PMC7483748; doi:10.1038/s42003-020-01233-4)
Supplement: Supplementary file 1 — Supplementary Information [file 42003_2020_1233_MOESM1_ESM.pdf]

Supplementary Figures

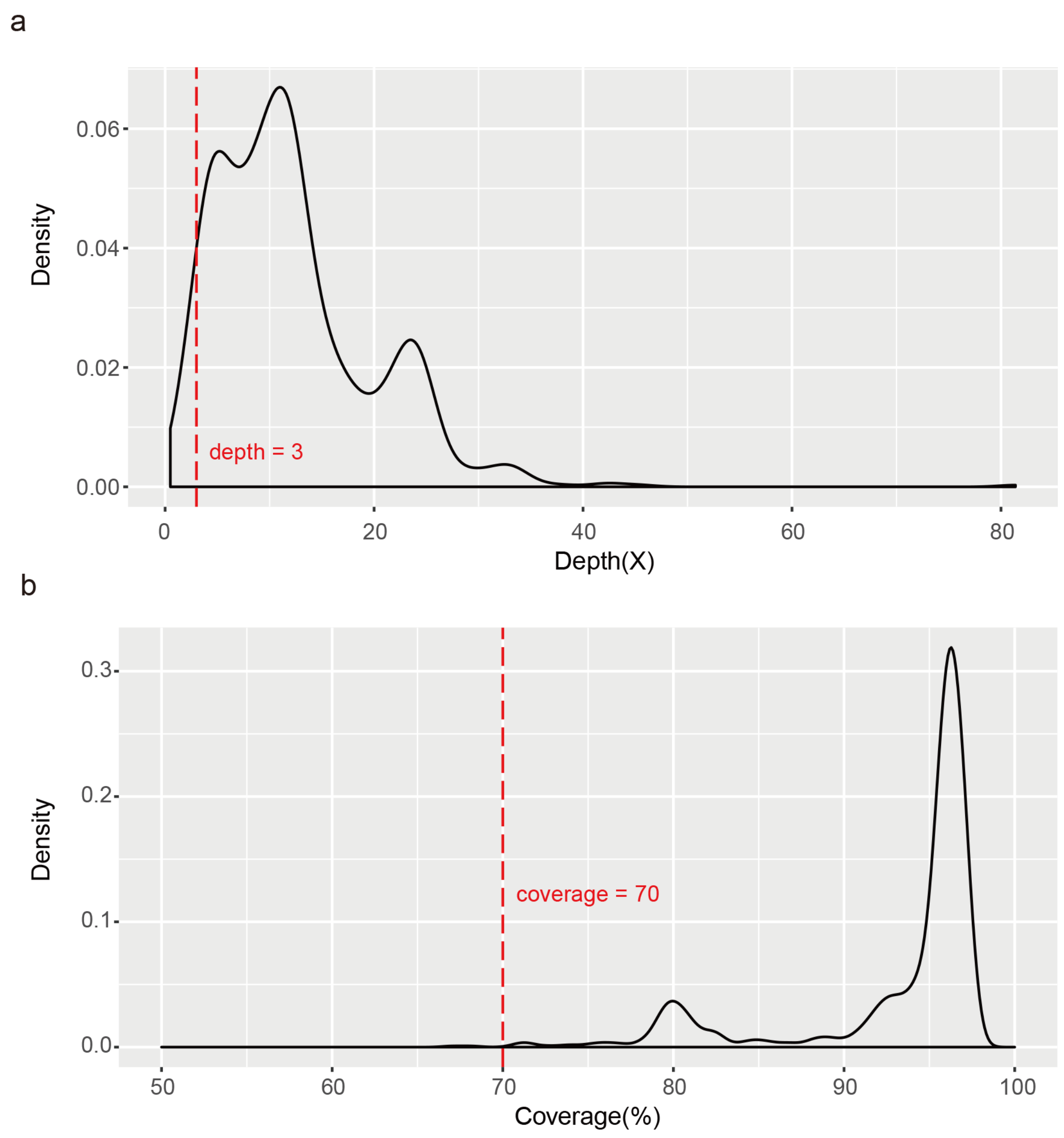

Supplementary Figure 1. Density distribution of sequencing depth and coverage in pig resequencing data. a. Density distribution of sequencing depth in swine resequencing data. b. Density distribution of sequencing coverage in resequencing data. Most data have a sequencing depth >3 and sequencing coverage >70%, and these samples are used to construct the omics knowledgebase.

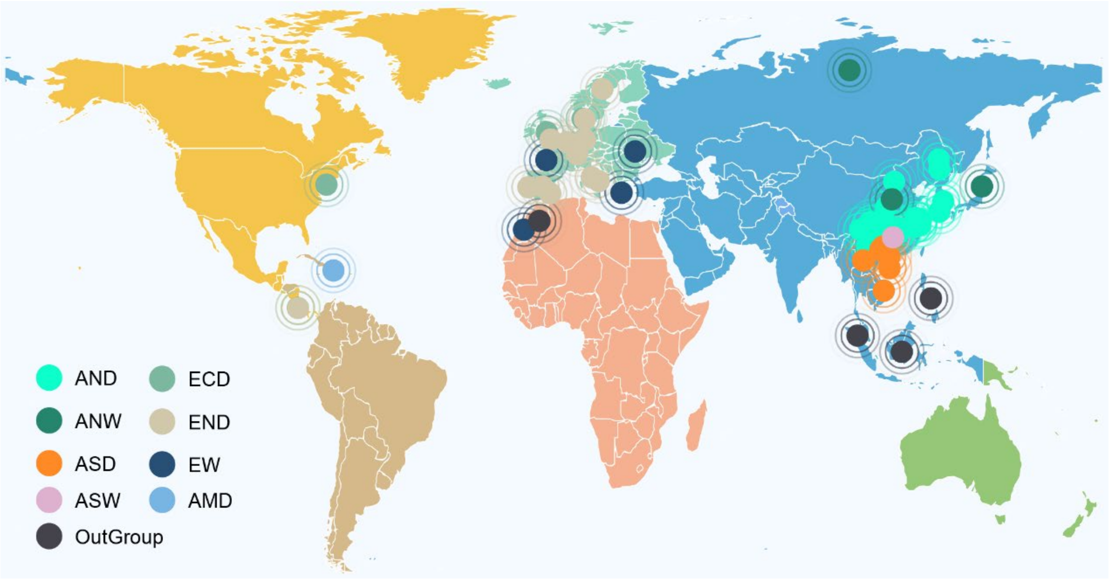

Supplementary Figure 2. Geographical distribution of the pig resequencing samples in the world. A total of 825 qualified individuals were retained for knowledgebase construction, which included 29 Asian native breeds, 20 European native breeds, three European commercial breeds, two American native breeds, and five other breeds (outgroup). AND: Asian northern domestic, ANW: Asian northern wild, ASD: Asian southern domestic, ASW: Asian southern wild, ECD: European commercial domestic, END: European native domestic, EW: European wild, AMD: America domestic, OutGroup: outgroup.

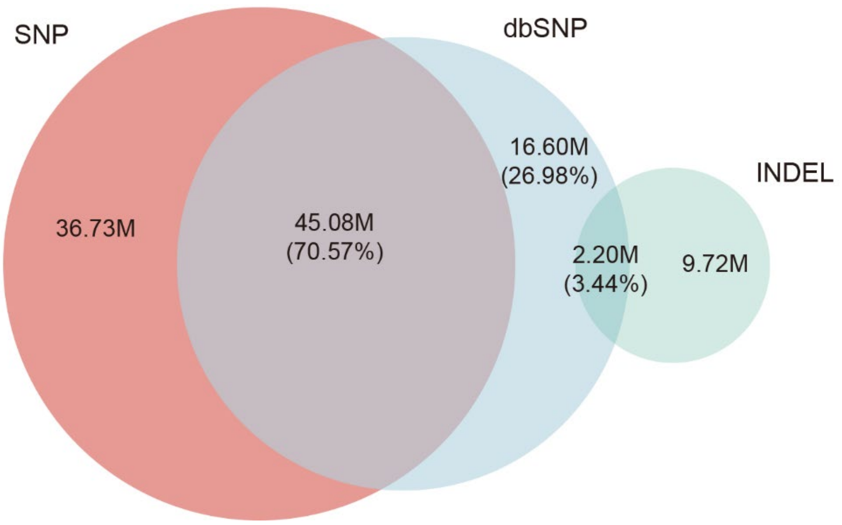

Supplementary Figure 3. Venn diagrams that show the distribution of shared and unique variations between ISwine and dbSNP. The dbSNP (Build 150) database from NCBI contains a great many pig variations and is a good reference to identify novel discovered variations. Our variant data set (both SNPs and indels) in ISwine covered >74.02% of its variants, and 46,451,715 variants were considered as novel.

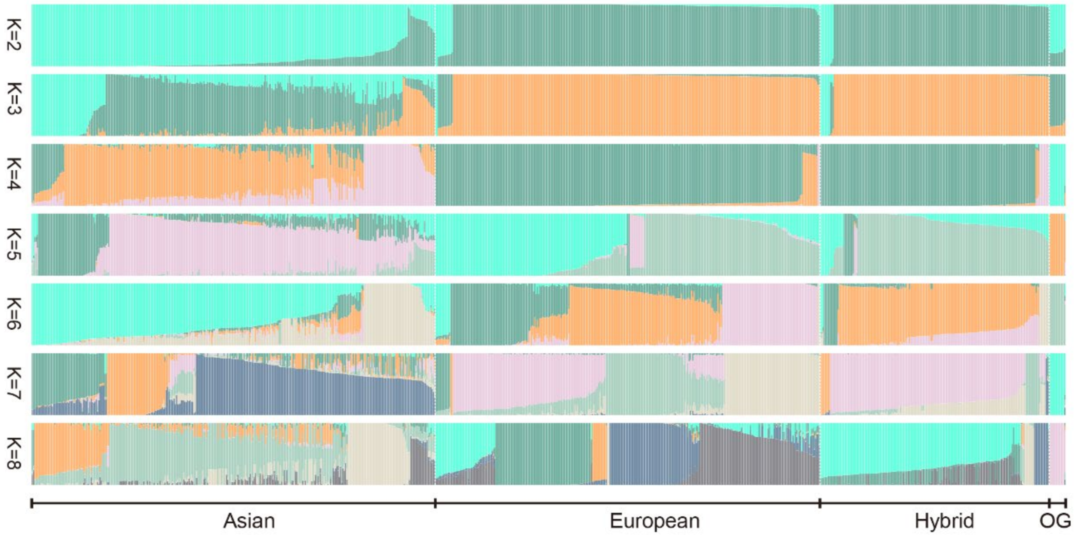

Supplementary Figure 4. Genetic structure analysis for 825 sequenced individual pigs (*Sus scrofa*) using ADMIXTURE with K = 2 to 8. Each individual was represented by a stacked column, which was partitioned into 2 - 8 colored segments with the length of each segment representing the proportion of the individual's genome from K = 2 - 8 ancestral populations. The first level of clustering (K = 2) reflected the primary geographical isolation between Asia and Europe. At K = 4, the outgroup (OG) became separated from Asian individuals.

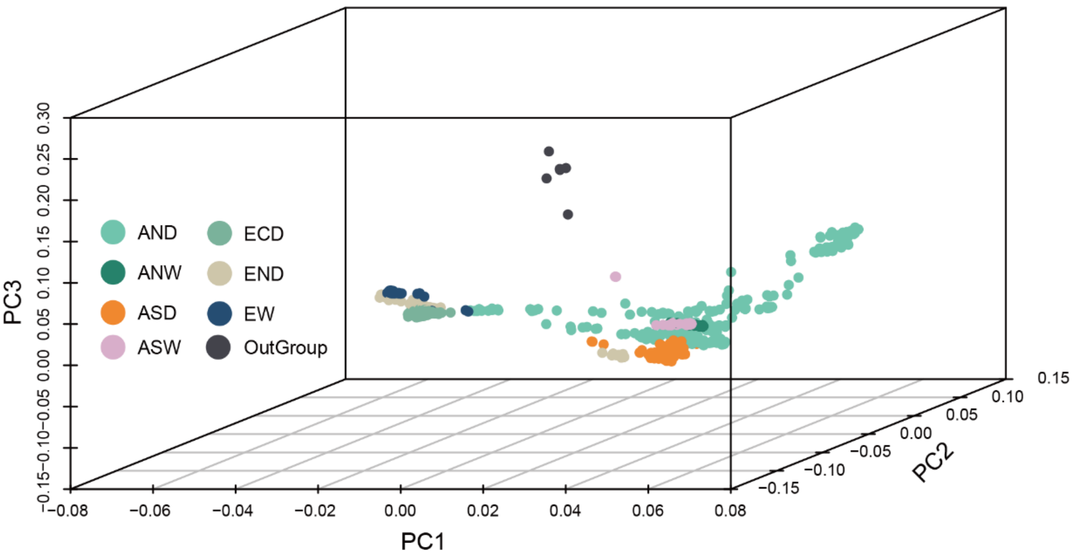

Supplementary Figure 5. Principal Component Analysis of all pig resequencing samples. The top three principal components were derived from the SNP genotype data and used for plotting the population structure. AND: Asian northern domestic, ANW: Asian northern wild, ASD: Asian southern domestic, ASW: Asian southern wild, ECD: European commercial domestic, END: European native domestic, EW: European wild, OutGroup: outgroup.

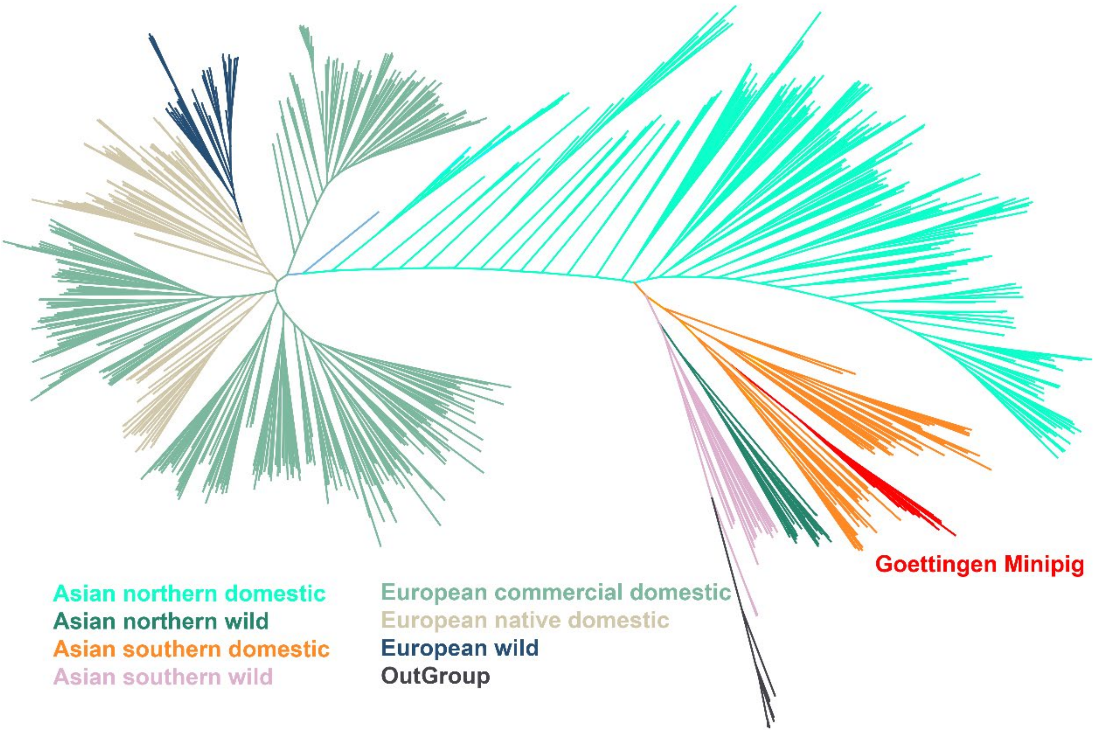

Supplementary Figure 6. Analysis of the phylogenetic relationship of all pig resequencing samples. The Asian and European pigs defined their own separate clades, and each clade split into a domesticated clade and a wild clade. The European Gottingen Minipig showed more genomic similarity to Asian southern pigs than to European native breeds.

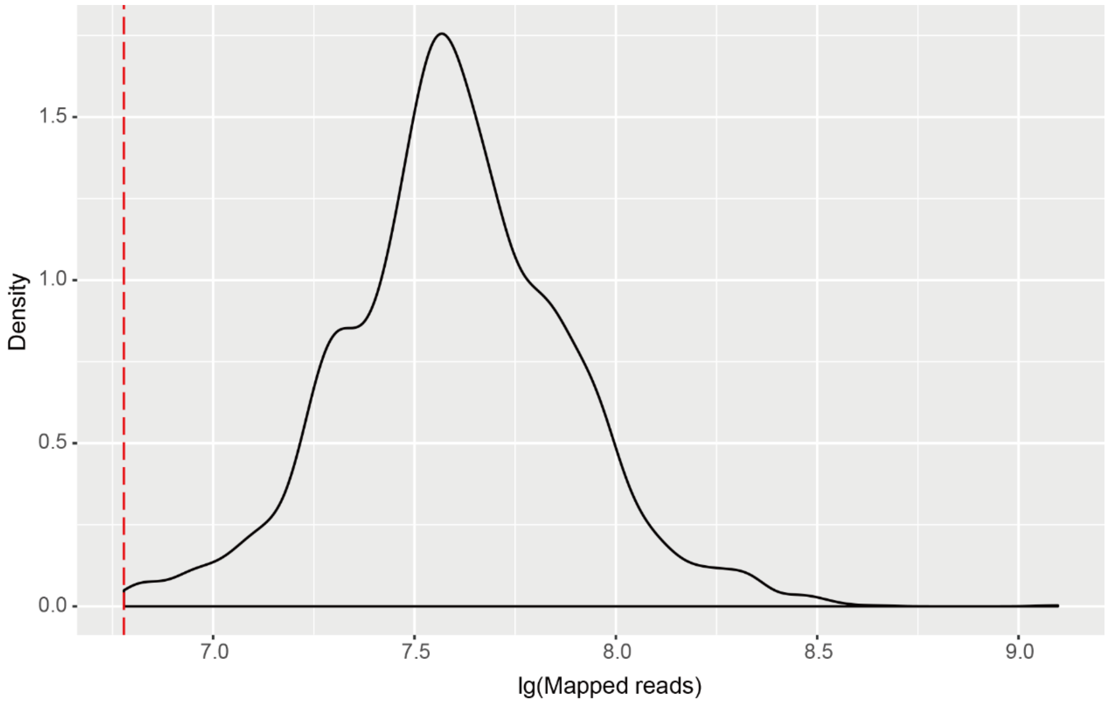

Supplementary Figure 7. Density distribution of mapped read counts in pig RNA-seq samples. The x-axis represents the logarithm of the mapped reads, and although we retained individuals with mapped reads > 6MB (dotted line) for knowledgebase construction, the mapped reads of the vast majority of samples were >10MB (7.0).

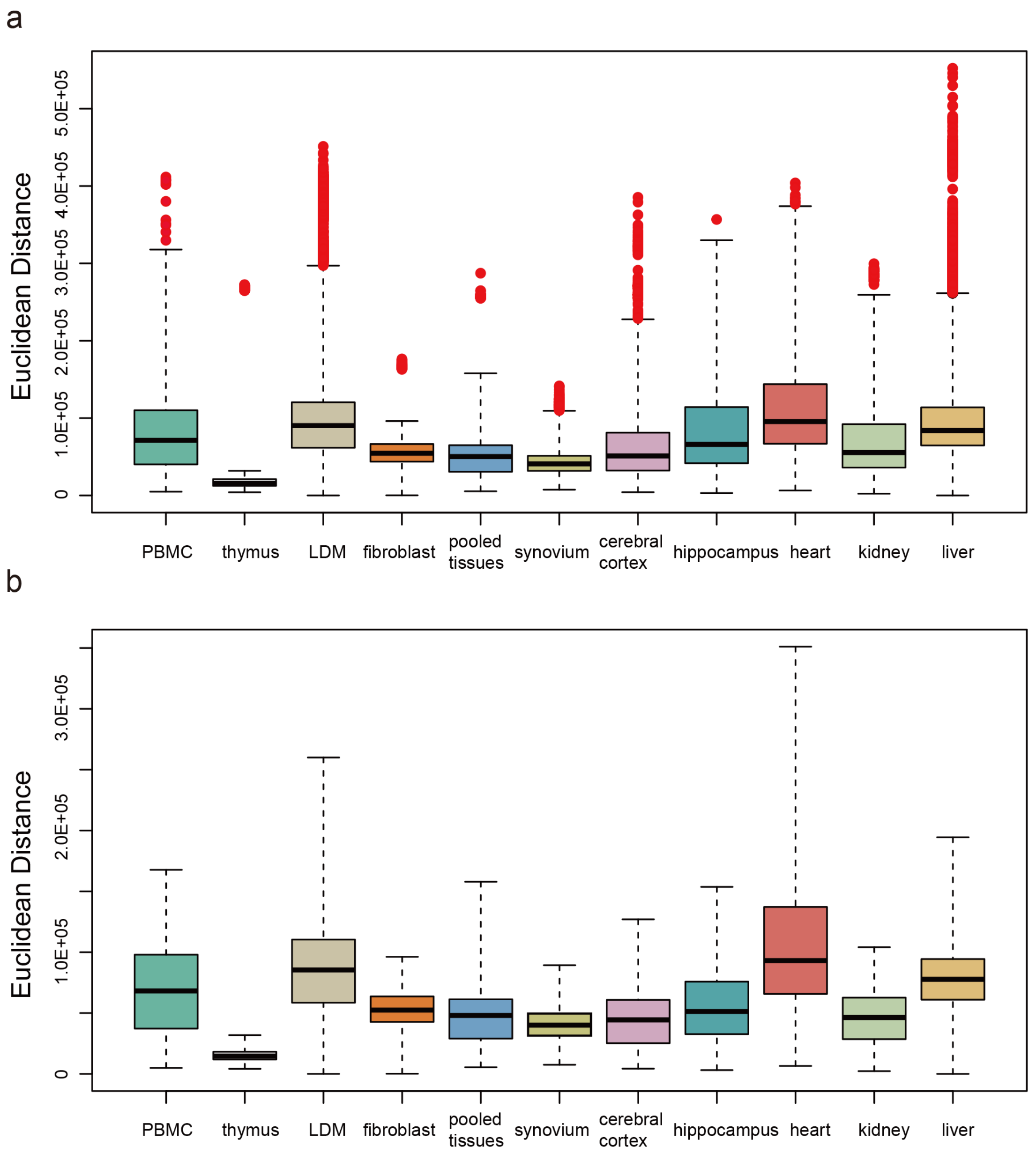

Supplementary Figure 8. Detection of discrete samples in various tissues of pigs. a. Euclidean Distance of the samples before removing the discrete samples. b. Euclidean Distance of the samples after removing the discrete samples. PBMC, Peripheral blood mononuclear cell; LDM, Longissimus Dorsi Muscle. The boxes denote the interquartile range (IQR) between the first and third quartiles, and the line inside denote the median. The whiskers denote the lowest and highest values within 3 times IQR from the first and third quartiles, respectively. Outliers beyond the whiskers are shown as red dots.

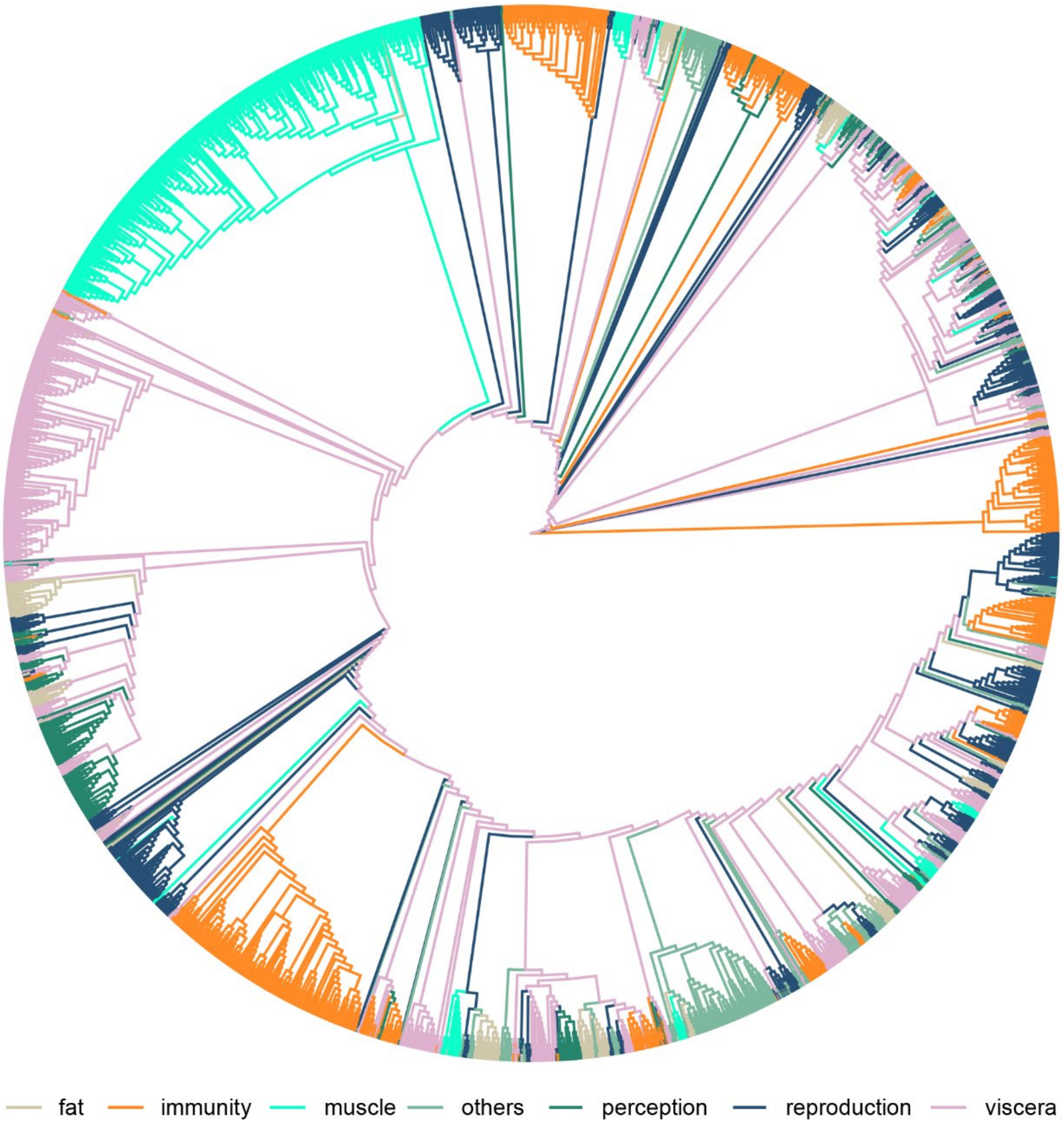

Supplementary Figure 9. Cluster analysis of pig RNA-seq samples in ISwine database. Most samples were grouped together in the tissue classification, but a small number of samples were discrete. It may have been related to the temporal and spatial specificity of the tissues or the sample collection method.

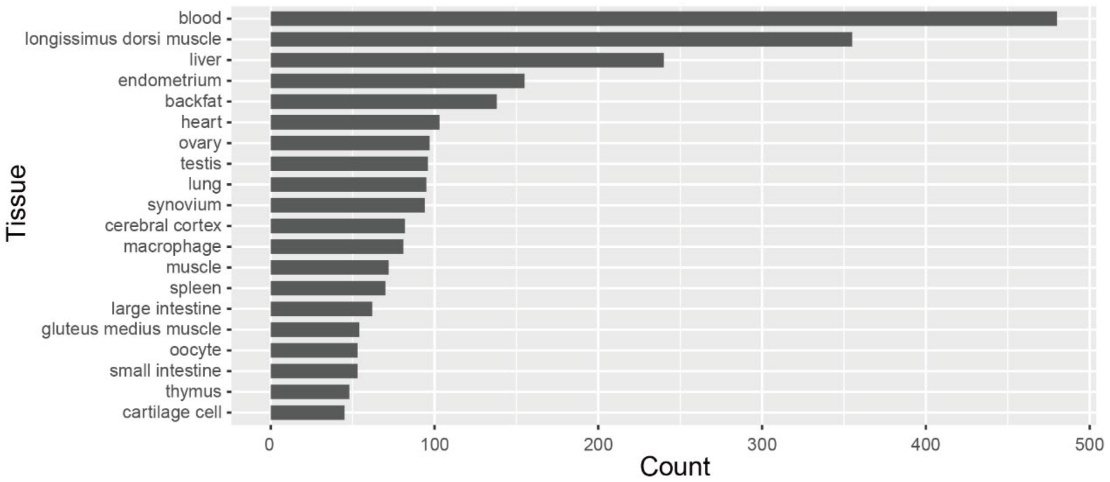

Supplementary Figure 10. Top 20 tissues of pigs in ISwine database. The x-axis represents the number of samples and the y-axis represents the tissues. The pig RNA-seq samples were mainly concentrated in the blood and longissimus dorsi muscle tissues. The liver, endometrium, back fat, and heart tissues also had a sample size >100.

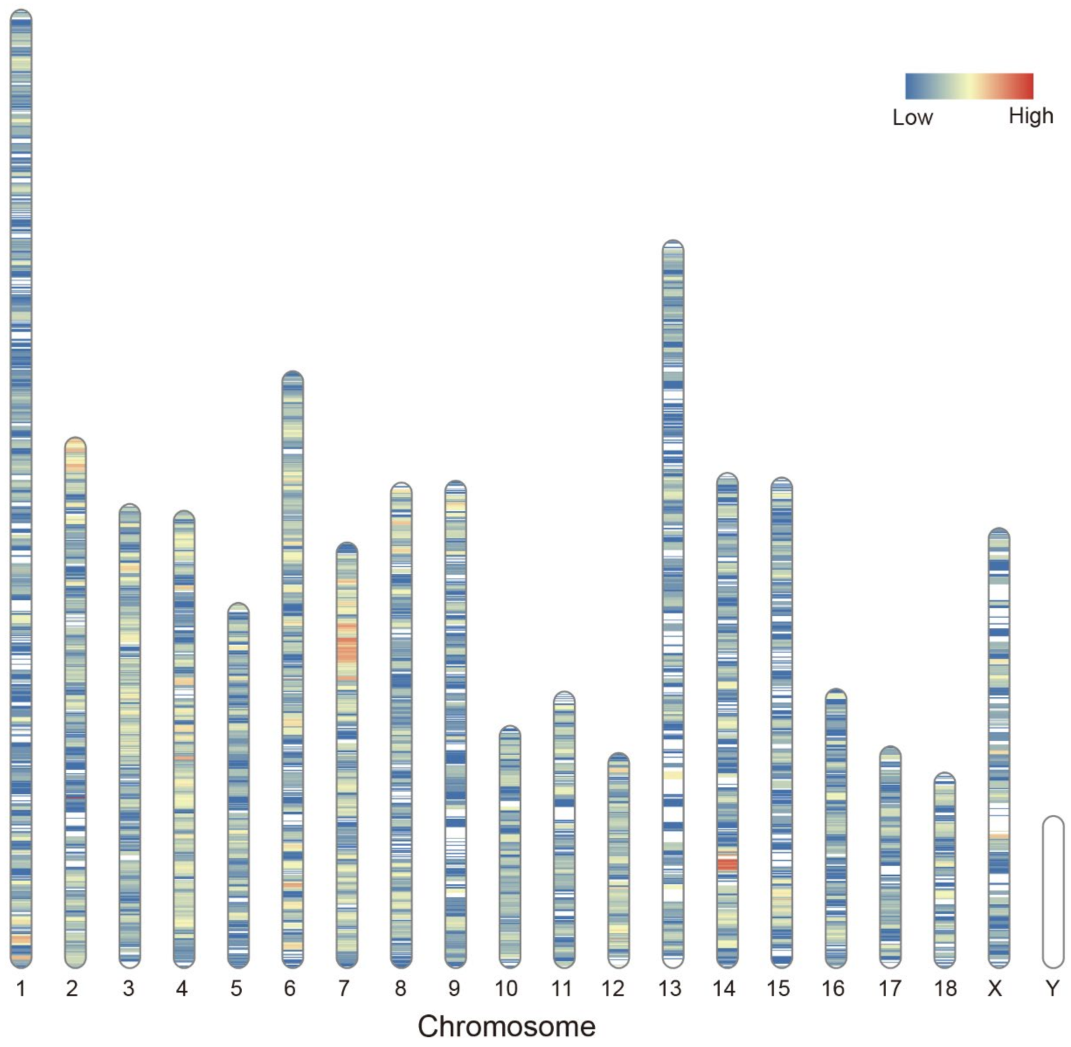

Supplementary Figure 11. Distribution of pig QTXs on whole genome chromosomes. The blue-yellow-red colors represent low-medium-high density of QTXs, respectively, and the blank region represents where no QTXs exist. The pig QTXs were distributed over the whole genome with the exception of chromosome Y.

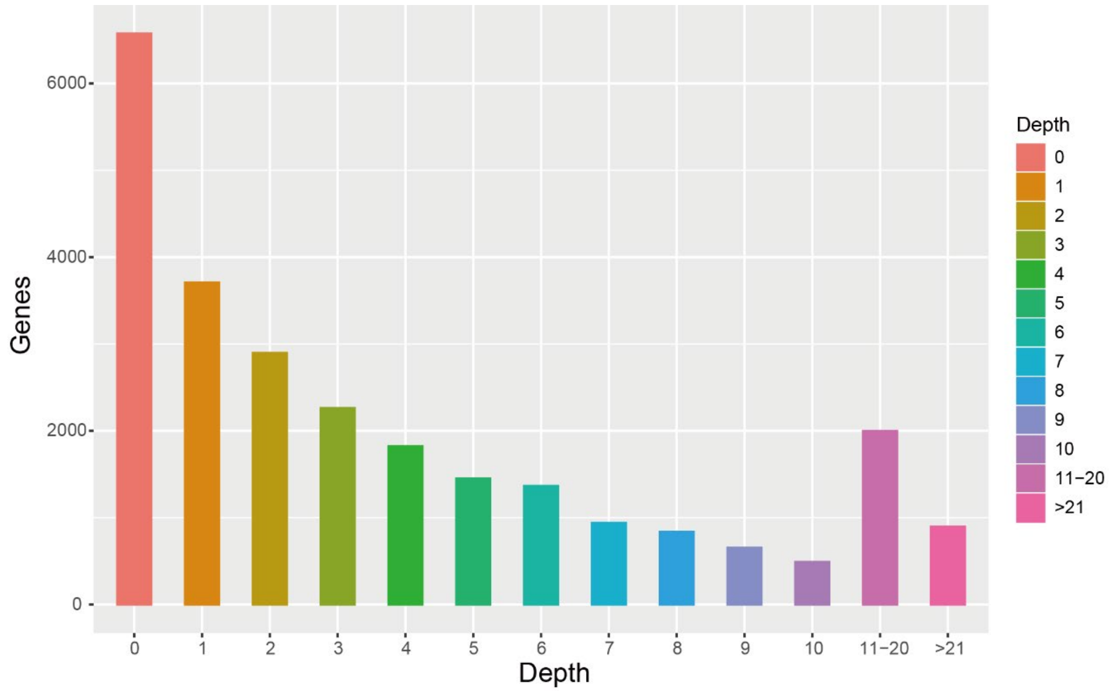

Supplementary Figure 12. The statistics of depth of coverage of QTXs for all genes in the swine genome. The x-axis represents the QTX depth, and the y-axis represents the number of genes. The QTXs covered 74.59% of the total genes, and most genes have low coverage of QTXs.

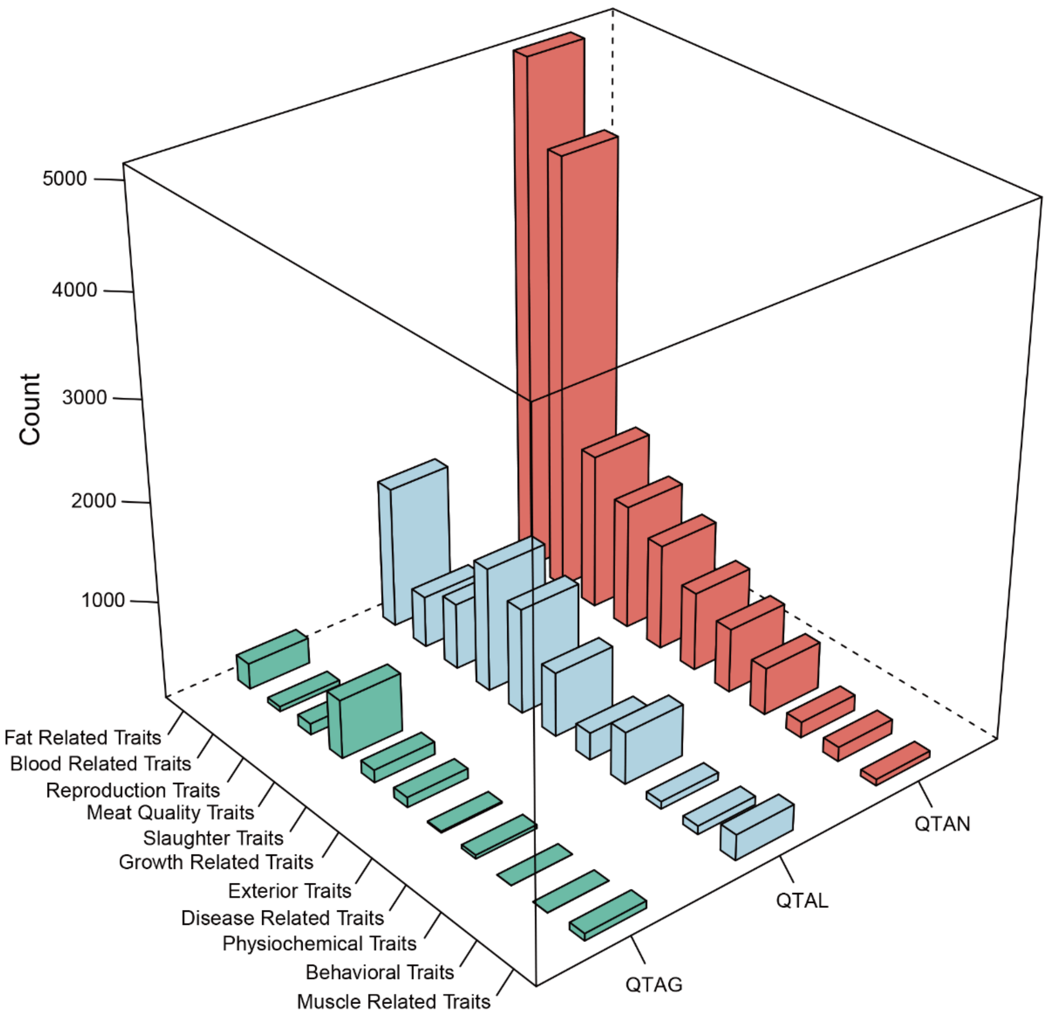

Supplementary Figure 13. Histograms of QTAG, QTAN, and QTAL in 11 QTX categories. The x-axis represents the trait categories of QTX, the y-axis represents the type of QTX, and the z-axis represents the number of QTXs. The relevant traits of 24,238 QTXs were divided into 11 major categories, and these QTXs, especially QTANs, were concentrated mainly in the "Fat Related Traits" and "Blood Related Traits" categories.

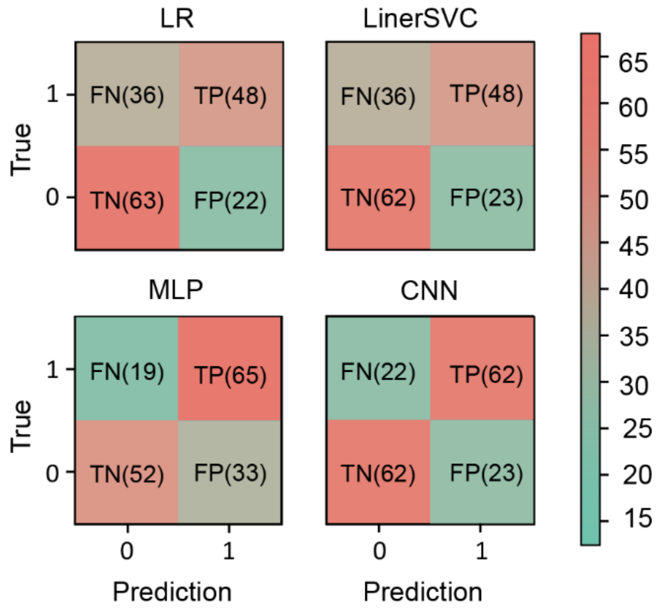

Supplementary Figure 14. Confusion matrix of four models in construction of the gene prioritization model. Each column of the matrix represents the instances in a predicted class, but each row represents the instances in an actual class. This makes it easy to see if the system is confusing two classes. LR: Logistic regression, LinearSVC: Linear Support Vector Classifier, MLP: Multi-Layer Perceptron, CNN: Convolutional Neural Networks, FN: False negative, TP: True positive, TN: True negative, FP: False positive.

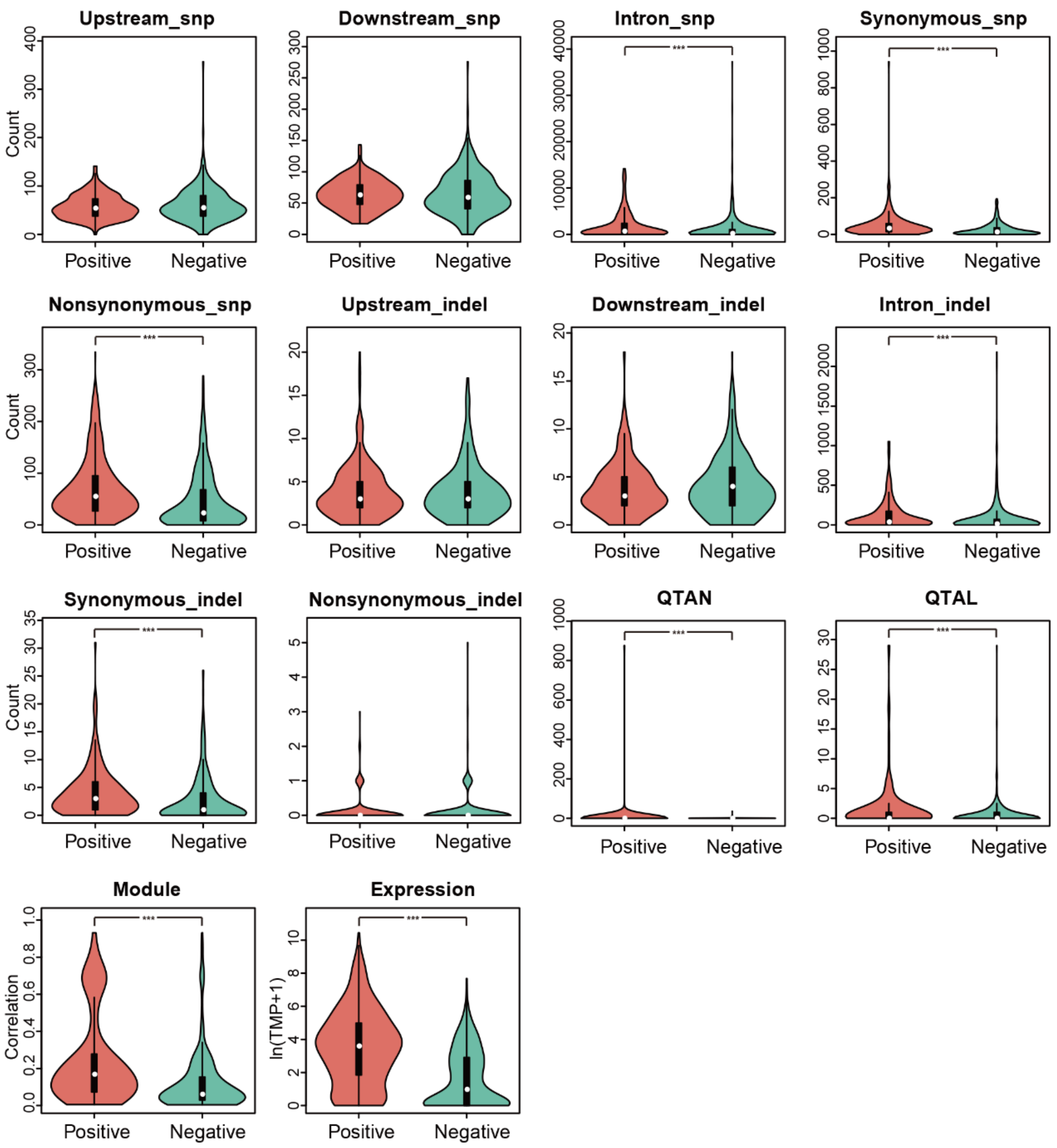

Supplementary Figure 15. The comparison of 14 features in positive and negative samples. Nine of the 14 features showed significant differences between positive and negative samples. The statistical significance was calculated by the Mann-Whitney test. ***: $P < 0.01$.

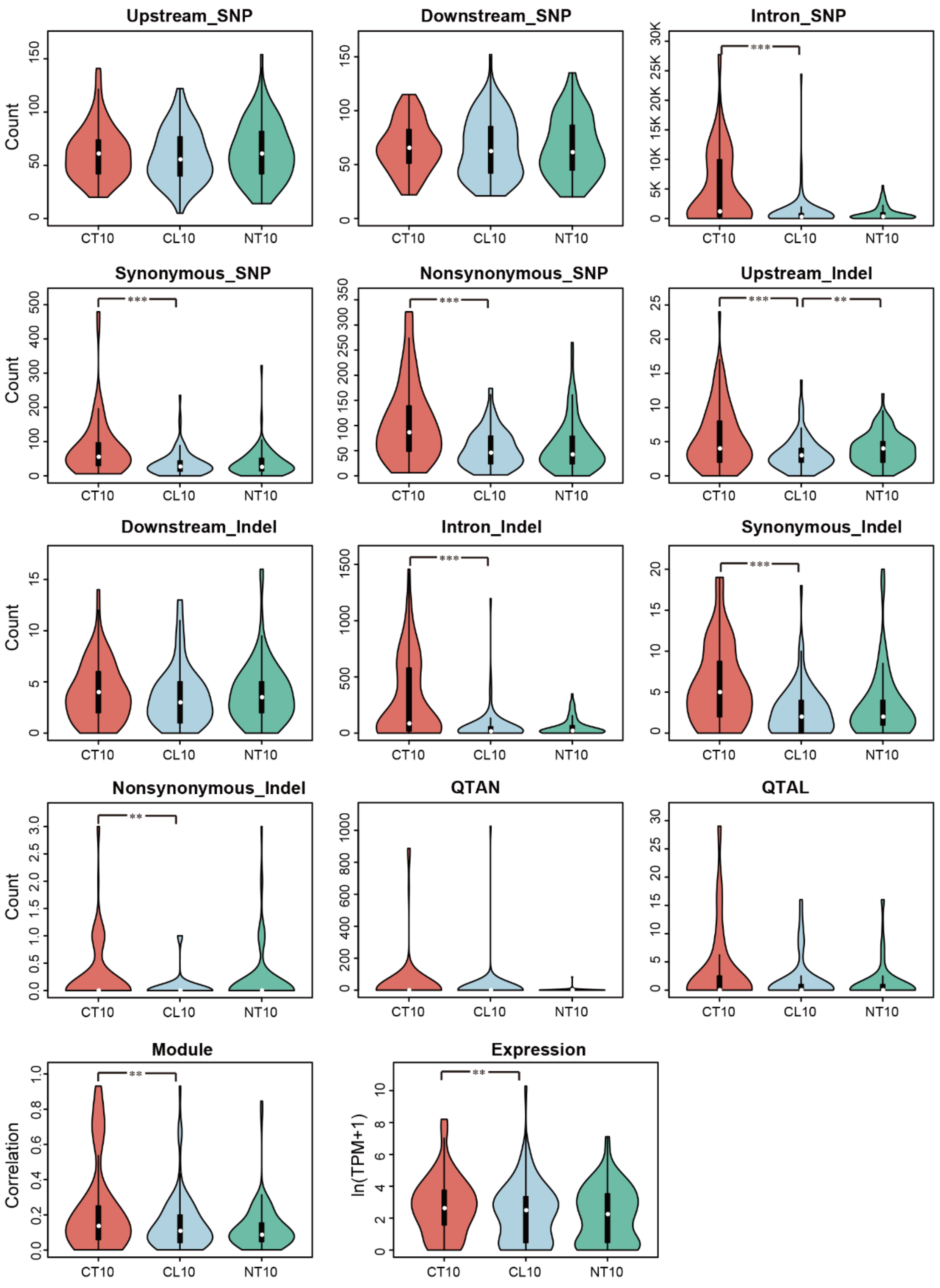

Supplementary Figure 16. Discovery of features characteristic of the candidate genes. Nine of the 14 features showed significant differences among three groups (CT10, CL10, and NT10) of genes, and six of the 14 features exhibited changing trends in the three groups. The statistical significance was calculated by the Mann-Whitney test. **: $P < 0.05$, ***: $P < 0.01$; CT10: top 10 of credible candidate genes, CL10: last 10 of credible candidate genes, NT10: top 10 of non-credible candidate genes.

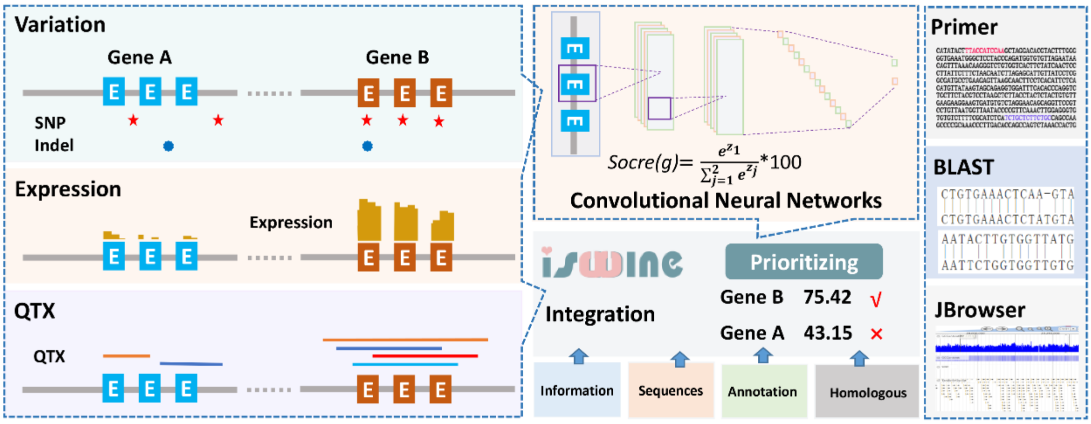

Supplementary Figure 17. Overview of the ISwine Knowledgebase. The left panel shows three basic databases, which include a variation database, an expression database, and a QTX database. The middle panel shows the multi-omics integration database (bottom) and a computing framework for gene prioritization (top). The integration database invokes information from basic databases and is combined with information about genes, (e.g., basic information, sequences, annotation, and homologous genes) to prioritize the genes of interest by using a CNN model. The right panel exhibits the tools embedded in ISwine.

Supplementary Tables

Supplementary Table 1. Summary of the downloaded pig genome data.

Project ID	Samples	Data Size(GB)	Valid samples	Valid Data Size(GB)
PRJEB1683	77	1,570.40	77	1,570.40
PRJEB2068	1	16.82	1	16.82
PRJEB27654	13	511.25	13	511.25
PRJEB9115	8	113.03	7	102.65
PRJEB9326	18	644.60	18	644.60
PRJEB9922	101	3,609.48	101	3,609.48
PRJNA144099	1	51.33	1	51.33
PRJNA176189	1	195.07	1	195.07
PRJNA176478	8	231.32	7	205.41
PRJNA186497	49	746.81	49	746.81
PRJNA190683	1	8.78	1	8.78
PRJNA213179	69	4,712.54	69	4,712.54
PRJNA221763	3	36.29	3	36.29
PRJNA231897	6	143.91	6	143.91
PRJNA238851	5	165.81	5	165.81
PRJNA239399	4	179.27	4	179.27
PRJNA240950	2	23.72	2	23.72
PRJNA254936	14	602.39	14	602.39
PRJNA255085	6	220.17	5	178.41
PRJNA260763	70	2,799.74	70	2,799.74
PRJNA273907	2	60.46	2	60.46
PRJNA281548	10	249.86	9	246.31
PRJNA291011	1	104.05	1	104.05
PRJNA305081	11	359.47	11	359.47
PRJNA305975	31	481.39	29	465.37
PRJNA309108	9	448.45	9	448.45
PRJNA314580	3	174.23	3	174.23
PRJNA320525	9	311.92	9	311.92
PRJNA322309	8	364.08	8	364.08
PRJNA343658	72	2,827.48	51	2,587.59
PRJNA354435	1	74.80	1	74.80
PRJNA358108	1	119.87	1	119.87
PRJNA369600	7	217.45	7	217.45
PRJNA378496	71	1,639.80	60	1,549.30
PRJNA393920	7	127.05	7	127.05
PRJNA398176	24	1,559.60	24	1,559.60
PRJNA41185	2	65.13	2	65.13
PRJNA414091	97	4,750.21	96	4,677.24
PRJNA418771	1	104.74	1	104.74
PRJNA438040	1	41.77	1	41.77

PRJNA482384	29	1,254.58	29	1,254.58
PRJNA507853	10	961.76	10	961.76

A total of 32.88 TB of resequencing data was obtained from 42 projects. After filtering, 825 qualified individuals were retained for subsequent analyses.

Supplementary Table 2. Breeds of the downloaded pig genome data.

Continents	Classify	Breed	Samples	Breed	Samples
Asian	AND	Bamei	7	Min	15
		Baoshan	6	Neijiang	9
		Daweizi	1	Penzhou	3
		Meishan	48	Rongchang	12
		Enshi black	3	Songliao black pig	2
		Erhualian	5	Taihu	1
		Hetao	6	Tibetan	55
		Jiangquhai	4	Tongcheng	5
		Jinhua	12	Wannan Spotted	2
		Korean black pig	14	Wujin	3
		Laiwu	6	Ya'nan	3
		Leping Spotted	2		
	ASD	Bamaxiang	6	MiniLEWE	3
		Diannanxiaoer	31	Wuzhishan	8
		Luchuan	6	Xiang	4
	ANW	Wild boar	21		
	ASW	Wild boar	19		
	OutGroup	Bornean Bearded pig	1	Javan warty pig	2
		Celebes warty pig	1	Visayan Warty pig	8
Africa	OutGroup	Warthog pig	1		
American	AMD	Creole	2	Yucatan minipig	13
	AMW	Wild boar	2		
European	ECD	Duroc	78	Yorkshire	40
		Landrace	45		
	END	Angler Sattleschwein	2	Large Black	1
		Berkshire	15	Leicoma	1
		British Saddleback	2	Linderodsvin	1
		Bunte Bentheimer	1	Mangalica	5
		Calabrese	1	Middle White	2
		Casertana	2	Hampshire	5
		Chato Murciano	2	Nero Siciliano	2
		Cinta Senese	1	Iberian	9
		Gloucester Old Spot	2	Pietrain	20
		Goettingen Minipig	12	Tamworth	3
	EW	Wild boar	38		
-	Hybrid	Hybrid	183		

The 825 qualified individuals included 29 Asian native breeds, 20 European native breeds, three European commercial breeds, two American native breeds, and five other breeds. AND: Asian northern domestic, ANW: Asian northern wild, ASD: Asian southern domestic, ASW: Asian southern wild, ECD: European commercial domestic, END: European native domestic, EW: European wild, AMD: America domestic, AMW: America wild, OutGroup: outgroup.

Supplementary Table 3. Summary of annotation of variations in ISwine database.

Category	SNP	indel
intergenic	44,927,893	6,448,062
upstream	1,050,989	159,226
UTR5	178,901	24,023
exonic	1,136,672	37,309
intronic	31,266,970	4,564,006
UTR3	855,901	140,529
downstream	1,076,563	164,397
upstream; downstream	58,906	9,606
UTR5;UTR3	5,758	884
unannotated	1,255,558	372,923

A total of 81,814,111 SNPs and 11,920,965 indels were identified, of which 51.4 million were intergenic, 35.8 million were intronic, and 1.17 million were exonic.

Supplementary Table 4. Functional gene categories enriched for the genes with QTX coverage depth >30.

Term ID	Term description	*P* value (BH)	Involved gene number
map04370	VEGF signaling pathway	1.06E-02**	9
map04668	TNF signaling pathway	1.75E-02**	10
map04010	MAPK signaling pathway	1.79E-02**	18
map00970	Aminoacyl-tRNA biosynthesis	1.71E-02**	7
map05131	Shigellosis	8.19E-03***	11
map04933	AGE-RAGE signaling pathway in diabetic complications	1.32E-02**	11
map05212	Pancreatic cancer	1.76E-02**	8
map05200	Pathways in cancer	1.86E-02**	25
map05221	Acute myeloid leukemia	2.63E-02**	7
map05169	Epstein-Barr virus infection	2.66E-02**	16
map05218	Melanoma	2.74E-02**	7
map05145	Toxoplasmosis	3.36E-02**	10
map05219	Bladder cancer	3.75E-02**	5
map04621	NOD-like receptor signaling pathway	4.19E-03***	14
map04915	Estrogen signaling pathway	9.99E-03***	11
map04723	Retrograde endocannabinoid signaling	1.63E-02**	8
map04912	GnRH signaling pathway	1.89E-02**	10
map04728	Dopaminergic synapse	2.77E-02**	11
map04920	Adipocytokine signaling pathway	4.21E-02**	8

The *P* values were calculated using a Benjamini & Hochberg -corrected modified hypergeometric

test. Only the KEGG-pathways with a *P* value <0.05 were considered as significant and listed. **: $P < 0.05$, ***: $P < 0.01$.

Supplementary Table 5. The distribution of QTXs in the main categories of the QTX database.

Main category	QTAL	QTAN	QTAG
Behavioral Traits	96	156	12
Blood Related Traits	531	4375	60
Disease Related Traits	538	487	48
Exterior Traits	287	660	25
Fat Related Traits	1,441	5,148	273
Growth Related Traits	661	808	112
Meat Quality Traits	1,268	1,272	605
Muscle Related Traits	270	75	83
Physiochemical Traits	86	170	11
Reproduction Traits	679	1,584	122
Slaughter Traits	1,078	1,079	138

The QTXs were concentrated mainly in the "Fat Related Traits", "Blood Related Traits", and "Meat Quality Traits" categories, which was consistent with mainstream research on pigs.

Supplementary Table 6. The relative importance of 14 features used in the CNN model.

Features	Relative importance(%)
Nonsynonymous_indel	100.00
Intron_snp	89.02
Expression	80.13
Module	78.01
QTAL	69.26
Intron_indel	41.28
Synonymous_snp	39.81
Upstream_indel	32.97
Upstream_snp	31.68
Downstream_indel	31.49
Downstream_snp	28.73
Synonymous_indel	23.81
QTAN	11.18
Nonsynonymous_snp	7.28

Except for the top five features, the relative importance of other features was < 50%, and the top five features may have played important roles in gene prioritization.

Supplementary Table 7. Performances (F1- Measure) comparison of the integrated models and single omics models.

Omics	LinearSVC	SVC	MLP	CNN
genome	0.613	0.599	0.689	0.711
transcriptome	0.489	0.539	0.608	0.614
literature	0.367	0.367	0.460	0.347
multi-omics	0.623	0.612	0.701	0.730

The F1- Measure was used to measure the performance of the model, and the performance of multi-omics was better than that of single omics, and the method based on neural network was superior to the linear method. LR: Logistic regression; LinearSVC: Linear Support Vector Classifier; MLP: Multi-Layer Perceptron; CNN: Convolutional Neural Networks.

Supplementary Table 8. The mean values of 14 features in positive and negative samples.

Feature	Positive	Negative	*P*
Upstream_snp	57.33	60.44	3.89E-01
Downstream_snp	63.76	64.24	4.34E-01
Intron_snp	1,894.59	1,143.98	1.06E-14***
Synonymous_snp	48.13	25.38	5.88E-22***
Nonsynonymous_snp	71.03	46.24	7.26E-18***
Upstream_indel	3.82	3.84	9.06E-01
Downstream_indel	3.78	4.01	4.60E-01
Intron_indel	121.86	76.90	2.67E-15***
Synonymous_indel	4.15	2.69	2.47E-14***
Nonsynonymous_indel	0.11	0.12	8.43E-01
Module	0.25	0.13	1.02E-22***
Expression	3.49	1.60	1.53E-36***
QTAN	5.65	0.69	1.98E-12***
QTAL	1.66	0.59	2.12E-07***

Nine of the 14 features showed significant differences between two datasets. **: $P < 0.05$, ***: $P < 0.01$.

Supplementary Table 9. Nine cases selected for evaluating the gene prioritization model.

Trait	Candidate genes	credible candidate genes	PMID
Fatty acid composition	580	250	30584983
Meat ultimate pH	121	60	30815891
Average daily gain	94	29	30974885
Backfat thickness	331	108	30974885
Lean percent	311	130	30974885
Average daily gain	279	85	31024621
Number of born alive	533	190	31029102
Backfat thickness	132	23	28890999
Backfat thickness	256	83	28196480

Overall, 50.41%-82.58% of the candidate genes were predicted to be non-credible candidate genes, which greatly narrowed the scope of credible candidate genes.

Supplementary Table 10. Number of credible candidate genes identified in nine traits.

Group	Trait1	Trait2	Trait 3	Trait 4	Trait 5	Trait 6	Trait 7	Trait 8	Trait 9
CT10	6	5	6	5	6	4	3	2	5
CL10	1	2	2	2	3	3	3	4	1
NT10	1	0	2	2	1	0	2	1	1

The number of credible candidate genes in CT10 was much more than CL10 (P = 2.36×10-3), and the credible candidate gene number in CL10 was more than NT10 (P = 9.53×10-3). CT10: top 10 credible candidate genes; CL10: last 10 credible candidate genes; NT10: top 10 non-credible candidate genes.

Supplementary Table 11. The proportion of credible candidate genes in different scoring ranges.

Score	credible candidate gene	Total genes	Ratio (%)
<50	10	90	11.11
50,60	16	76	21.05
60,90	5	18	27.78
90,100	42	86	48.84

The proportion of credible candidate genes in different scoring ranges increased (from 21.05 to 48.84) with the gene score, which suggested that a candidate gene was reliable if its gene score was high enough.

Supplementary Table 12. The mean values of 14 features in CT10, CL10, and NT10 groups.

Feature	CT10	CL10	NT10	*P* (CT10_CL10)	*P* (CL10_NT10)
Upstream_snp	61.70	58.88	63.18	5.24E-01	3.38E-01
Downstream_snp	66.47	65.68	66.43	6.85E-01	8.32E-01
Intron_snp	5,048.96	1,018.84	855.94	9.82E-08***	2.56E-01
Synonymous_snp	81.52	36.03	37.88	7.66E-08***	6.23E-01
Nonsynonymous_snp	105.87	53.39	58.46	3.69E-07***	9.91E-01
Upstream_indel	5.62	3.19	3.89	1.00E-04***	1.38E-02**
Downstream_indel	3.94	3.62	3.84	3.49E-01	4.00E-01
Intron_indel	293.22	58.22	53.11	5.01E-08***	1.87E-01
Synonymous_indel	5.90	2.70	3.23	4.19E-07***	6.29E-01
Nonsynonymous_indel	0.24	0.07	0.17	1.28E-02**	1.88E-01
Module	0.23	0.15	0.13	4.04E-02**	2.91E-01
Expression	2.73	2.29	2.15	4.47E-02**	3.89E-01
QTAN	40.68	13.73	1.88	7.09E-02	1.04E-01
QTAL	3.39	1.84	1.34	3.19E-01	6.99E-02

Nine of the 14 features showed significant differences among three groups (CT10, CL10, and NT10) of genes, and six of the 14 features exhibited changing trends in the three groups. **: $P < 0.05$, ***: $P < 0.01$; CT10: top 10 credible candidate genes; CL10: last 10 credible candidate genes; NT10: top 10 non-credible candidate genes.

Supplementary Table 13. Statistics of the distance from credible candidate genes to the GWAS top signal.

Distance(KB)	credible candidate genes	Total genes	Ratio(Range)	Ratio(credible candidate genes)
0-200	25	71	34.25	35.21
200-400	15	57	20.55	26.32
400-600	11	48	15.07	22.92
600-800	7	44	9.59	15.91
800-1000	15	50	20.55	30.00

Candidate genes that were close to a GWAS peak signal had a higher proportion of credible candidate genes than those far away from the peak, but the proportion of credible candidate genes at near and far distances was similar, which indicated that distal regulation should be considered in the identification of credible candidate genes.

Supplementary Table 14. Comparison of swine variation databases.

Database	Individuals	Number of Variations	Individual genotype	Assembly Version
ISwine	825	93,735,076	Available	11.1
pigVar	280	71,819,600	Available	10.2
dbSNP	NA	63,881,778	NA	11.1
GVM	409	76,797,395	NA	10.2

Compared with existing swine databases, such as pigVar, dbSNP from NCBI (updates have stopped), and the Genome Variation Map (GCM), ISwine has the largest number of variations and number of resequencing individuals.

Supplementary Note 1: Database interface and general functions

ISwine has provided a user-friendly interface for users to browse, search, visualize, download, and analyze the structured omics data. A top navigation bar was designed to assist users to access the above-mentioned database ("Integration", "Variation", "Expression", and "QTX”) and to use the functions of the “BLAST”, “Primer”, “JBrowser”, and “Prioritize” tools. To facilitate the acquisition of information for users from the database, we designed various search modes based on the characteristics of different data, such as key field search mode, region search mode, and associated information search mode. The users can obtain their interested information flexibly by choosing an applicable mode.

In addition to search engines, we also offer different functions for different databases. These functions mainly emphasize the interaction and visualization of results, so users can get information of interest intuitively**.** For example, in the variation database, the user can construct one or more populations by sample attributes or sample individuals through the population design module (**Figure 3a**), so they can observe mutations in different populations easily. We also visualized the variations and related genes in the genome (**Figure 3b**), and the user can choose a SNP/indel quickly by clicking the site directly. Finally, users can obtain the genotypes of all individuals at their interested sites through the variation details page (**Figure 3b**). In addition, all results in the variation database are not only interactive online, but also downloadable, so users can select their favorite tools to filter and to analyze the data.

In the expression database, we display the gene expression level or fold change of differential expressed genes with heatmap (**Figure 3c**), so users can observe the expression characteristics among different samples. We also designed filter functions for users to adjust the number of samples or genes displayed in the expression matrix. In the transcriptome study, the tissue information of the sample has received much attention. We designed a gene expression profile module (**Figure 3d**) for users to observe gene expression patterns in different tissues. The gene expression profile module displays one or more genes with heatmaps, boxplots, and line graphs, and the user can adjust the number of genes displayed in the image by clicking a convenient

label.

In the QTX database, a physical map (**Figure 3e**) was constructed for users to select their QTXs in a convenient and intuitive way, and the positional relationship of QTX on the genome was reflected more intuitively. In addition, due to the low quality of some QTALs, we also designed a QTX rating system (**Figure 3f**) so the user can judge the authenticity of the QTAL more correctly by referring to the evaluations of other users.

The functions designed for the integration database mainly focus on data aggregation and integration. First of all, we provide an advanced search engine (**Figure 3a**) so that users can easily use the information of the basic database to filter the genes of interest. And secondly, we designed a gene prioritization model (**Figure 3c**) so users can easily use this system to prioritize the genes of interest by inputting a gene list or region list. The system will return a sorted gene table, and the user can further access the information on gene details (**Figure 3b**) that are aggregated by database to finally determine the credible candidate genes and then to mark and to export them from the gene table. For the gene details page, we added some simple and practical functions, such as copying of sequences, display of structures, and more functions are called directly from the basic database ("Variation", "Expression", and "QTX”).

Finally, we provide users with some downstream tools to analyze credible candidate genes, such as Primer (**Figure 3k**) for primer designing, BLAST (**Figure 3l**) for sequence targeting, and JBrowser (**Figure 3k**) for visualizing genetic components.

Supplementary Method 1: Evaluation Metrics. The averages of model accuracy, precision, recall, and F1 scores were calculated to evaluate the model’s performance by using a 4-fold cross-validation method. The model accuracy was defined as the ratio between the number of samples identified correctly and total number of samples in the training set. The model precision was defined as the ratio between the number of positive samples identified correctly and total number of positive samples in the training set. The model recall was defined as the ratio between the number of genes identified correctly and total number of identified genes, and the model F1 score was

the harmonic mean of precision and recall:

$$Accuracy = \frac{True\ \ positive\ \ samples + True\ \ negative\ \ samples}{Total\ \ samples\ \ in\ \ training\ \ set}$$

$$Recall = \frac{True\ \ positive\ \ samples}{Total\ \ positive\ \ samples\ \ in\ \ training\ \ set}$$

$$Precision = \frac{True\ \ positive\ \ samples}{Total\ \ positive\ \ samples\ \ predicted}$$

$$F1 = \frac{2 * Precision * Recall}{Precision + Recall}$$
