# Supplementary material for: A gene prioritization method based on a swine multi-omics knowledgebase and a deep learning model
Source: Commun Biol. 2020 Sep 10;3:502. doi: 10.1038/s42003-020-01233-4 (PMC7483748; doi:10.1038/s42003-020-01233-4)
Supplement: Supplementary file 2 — Description of Additional Supplementary Files [file 42003_2020_1233_MOESM2_ESM.pdf]

Description of Additional Supplementary Files

Supplementary Data 1. Summary and mapping statistics of genome data in ISwine database.

Supplementary Data 2. Summary of the downloaded pig transcriptome data. A total of 20.0 TB of sequencing data, 263 projects, and 3,526 samples were downloaded and analyzed.

Supplementary Data 3. Summary and mapping statistics of transcriptome data in ISwine database.

Supplementary Data 4. Tissue classification and statistics of pig transcriptome data in ISwine database. A total of 3,282 samples were classified into seven main categories and 95 subcategories based on tissue classification and their relative position in the swine's body.

Supplementary Data 5. The list of QTXs related literatures in ISwine database.

Supplementary Data 6. The information of the trimmed QTALs.

Supplementary Data 7. The statistics of QTXs coverage depth for all genes in the swine genome.

Supplementary Data 8. Trait classification and statistics of pig QTX data in ISwine database. The relevant traits of 24,238 QTXs in pig were divided into 11 major categories and 89 subcategories.

Supplementary Data 9. The Training Set used in this study.

Supplementary Data 10. The details of positive samples used in this study.

Supplementary Data 11. References to credible candidate genes in different trait cases. The green, pink, and gray backgrounds represent the candidate gene is reported to be credible candidate in the case literature, in other literatures, and non-reported, respectively.
